# Factors Associated With Professionals’ Context-Specific Trust in AI-Based Screening for Autism Spectrum Disorder: Cross-Sectional Survey Study

**DOI:** 10.2196/98367

**Published:** 2026-09-25

**Authors:** Yoshimasa Ohmoto, Kazunori Terada, Ryoichiro Iwanaga, Hirokazu Kumazaki

**Affiliations:** 1 Department of Behavior Informatics, Faculty of Informatics, Shizuoka University, Shizuoka, Hamamatsu, Japan; 2 Department of Electrical, Electronic, and Computer Engineering, Faculty of Engineering, Gifu University, Gifu, Japan; 3 Unit of Medical Science, Nagasaki University Graduate School of Biomedical Sciences, Nagasaki, Japan; 4 Department of Neuropsychiatry, Graduate School of Biomedical Sciences, Nagasaki University, 1-7-1, Sakamoto, Nagasaki, 852-0052, Japan, 81 958197291, 81 958197296

**Keywords:** artificial intelligence, AI, autism spectrum disorder, screening, AI-based diagnosis, human-AI collaboration, decision-making, risk assessment, AI adoption, AI skepticism

## Abstract

**Background:**

In clinical settings, clinicians’ context-specific trust in AI is associated with individual factors. This is particularly relevant in autism spectrum disorder (ASD) screening, where diagnostic criteria are complex, heterogeneous, and inherently uncertain. Although some AI systems demonstrate high sensitivity, clinicians are not always convinced of their utility. Therefore, examining potential biases in context-specific trust and achieving well-calibrated trust are critical.

**Objective:**

This study aimed to examine the individual factors associated with professionals’ context-specific trust in AI-assisted ASD screening systems.

**Methods:**

Fifty-five professionals involved in ASD assessment participated in the study. We used an AI-based ASD screening method based on a geometric shape-drawing task that assesses children’s motor function and has demonstrated high sensitivity for identifying children with ASD. Expert clinicians selected 2 ambiguous screening scenarios for evaluation. Participants made independent judgments while considering the AI output and rated their context-specific trust in each decision on a 7-point Likert scale. A linear mixed model (LMM) examined the associations between context-specific trust in AI decisions and participant characteristics while accounting for the nested data structure.

**Results:**

LMM analysis revealed significant between-participant variability in context-specific trust. Years of professional experience (*t*_46.3_=−2.20; *P*=.03) and the length of education required to obtain professional credentials (*t*_47.6_=−2.67; *P*=.01) were significantly and negatively associated with context-specific trust in AI-based screening. Dispositional trust in AI was significantly and positively associated with context-specific trust (*t*_46.1_=2.78; *P*=.008). Confidence in participants’ own assessments was significantly and positively associated with context-specific trust (*t*_91.0_=5.67; *P*<.001).

**Conclusions:**

Our findings suggest that more experienced clinicians tend to exhibit greater skepticism toward AI, consistent with observations in other medical fields such as oncology. To support the effective integration of AI into clinical practice, professional education should include training on AI principles and strategies for appropriate human-AI collaboration. Future studies with larger and more diverse samples, multimodal assessment approaches, and real-world clinical settings are needed to validate these findings and inform the implementation of AI-assisted ASD screening.

## Introduction

In medicine, hybrid decision-making by professionals in collaboration with AI is recognized as a powerful approach to enhancing decision-making [1-4]. However, its effectiveness depends on appropriate trust in AI systems, as both overreliance and underreliance may impair clinical decision-making [5,6]. To achieve well-calibrated trust, professionals must understand their own capacities as well as the capabilities and limitations of AI systems and recognize potential biases in their trust [7]. In medicine, trust in AI is associated with individual factors, including personality traits [8,9], demographic characteristics [10], and experience with AI [11]. This issue is particularly relevant in domains such as autism spectrum disorder (ASD) screening, where the diagnostic criteria are complex, heterogeneous, and inherently uncertain.

ASD is a complex neurodevelopmental condition characterized by deficits in social communication and interaction, as well as restricted and repetitive behaviors [12]. According to the United States Centers for Disease Control and Prevention, approximately 1 in 36 children is diagnosed with ASD [13]. The lifetime social cost associated with ASD is estimated at US $3.6 million per individual [14].

Early and accurate screening is crucial for timely intervention, which can significantly improve developmental outcomes and parental support [15,16]. However, ASD screening remains challenging because of its highly variable presentation and overlap with other neurodevelopmental disorders [17]. Most screening tests are questionnaire-based and demonstrate low to moderate accuracy [18]. Furthermore, they are prone to recall and subjectivity biases [19]. These limitations underscore the need for more objective and reliable approaches, including data-driven or technology-assisted methods.

AI-based tools for screening children with ASD are currently being developed worldwide. Some systems demonstrate high sensitivity in identifying children with ASD [20-24]. However, these systems are based primarily on statistical correlations, and their outputs require careful interpretation. Additionally, results are often presented in complex, multidimensional forms, which may limit their interpretability and usability in clinical decision-making. Despite their potential, clinicians are not always convinced of the utility of AI-based screening tools, particularly in ASD, where concerns persist regarding the empirical validity and standardization of existing approaches [25]. These challenges highlight the importance of understanding how clinicians perceive and trust AI systems in ASD screening.

Given the complexity of ASD diagnostic criteria and the multidimensional nature of AI outputs, the individual factors influencing professionals’ trust in AI-based ASD screening may differ from those in other medical domains. Therefore, examining potential biases in trust and achieving well-calibrated trust is essential. Following prior work distinguishing context-specific trust from actual trusting behavior [26,27], we define trust in this study as a professional’s rated trust in the AI system’s clinical decisions. In this study, professionals viewed videos of an inverted equilateral triangle drawing task alongside the corresponding AI-based assessment process and rated their trust in the AI system’s assessment. The aim of this study was to examine professionals’ context-specific trust in AI-assisted ASD screening and the individual factors associated with it.

## Methods

### Participants

Fifty-five ASD assessment professionals participated in this study. They were recruited from 4 developmental support institutions in Japan through email invitations sent to institutional contacts. Sampling was expanded through professional networks. A total of 55 eligible professionals were approached, and all agreed to participate, resulting in a 100% participation rate. The study used a within-participant design in which each participant completed 2 assessment trials, yielding 110 total observations.

### Ethical Considerations

The study was approved by the Medical Review Board of Gifu University Graduate School of Medicine (2025‐257). All procedures involving human participants were conducted in accordance with the ethical standards of the institutional and/or national research committee, the 1964 Declaration of Helsinki and its later amendments, and comparable ethical standards. All participants provided informed consent electronically before completing the survey. All data were anonymized before analysis, and no personally identifiable information was collected. Data were securely stored and accessible only to the research team. Participants received no financial compensation. This study was reported in accordance with the STROBE (Strengthening the Reporting of Observational Studies in Epidemiology) statement for observational studies [28] and the CHERRIES (Checklist for Reporting Results of Internet E-Surveys) for web-based surveys [29].

### Procedures

This study was conducted in a web-based setting using an online survey platform. Participants were recruited from established developmental support institutions in Japan, with recruitment facilitated by institutional directors. The inclusion criterion was that participants’ primary professional role involved assessing children with ASD. Participants accessed the study through a secure web link and completed all study procedures. The study session comprised 3 phases: a demographic questionnaire, video-viewing with assessment tasks, and a postviewing questionnaire.

We used an AI-based ASD screening method centered on a geometric shape-drawing task [21]. The AI system used a support vector machine with a linear kernel, trained on 16 variables extracted from children’s pen kinematics and eye movement patterns during an inverted equilateral triangle drawing task. In previous validation studies, this model demonstrated approximately 90% sensitivity and 100% specificity [21]. We presented the AI system’s judgment, generated by this framework, as the AI output in the experimental task. In this study, expert clinicians selected 2 diagnostically challenging cases to represent ambiguous screening scenarios.

Participants first completed a demographic questionnaire that collected information on age, sex, profession, years of professional experience, educational background, length of education required to obtain professional credentials, and frequency of AI application use. AI application use was originally rated on a 5-point scale from 0 (never) to 4 (daily) and dichotomized for analysis (0=never or rarely, 1=several times per month, several times per week, or daily). This categorization reflects a qualitative threshold distinguishing sporadic interaction from regular, integrated workflow use.

**Figure 1.**
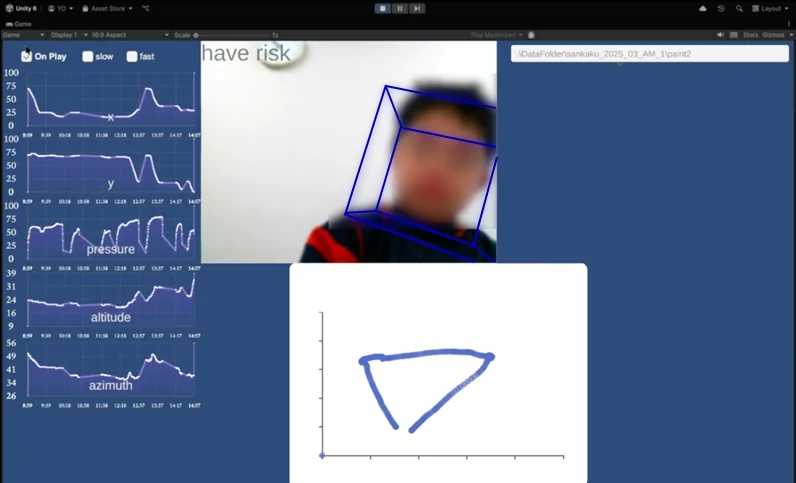
Screenshot of a video of a child performing an inverted equilateral triangle drawing task on a digital tablet. The left panel shows time-series graphs of drawing characteristics, including x- and y-position coordinates (0‐100 scale), pen pressure (0‐100 scale), pen altitude angle (14°‐44°), and pen azimuth direction (20°‐50°). The top middle panel displays a video feed of the child, including facial expressions and gaze features. The bottom middle panel shows the drawing in real time. Each video also displays the AI system’s risk assessment, categorized as either “low risk” (low ASD risk) or “high risk” (ASD risk present).

In the video-viewing phase, participants watched 2 cases in sequence. Each video presented a child drawing an inverted equilateral triangle on a digital tablet. The videos were professionally produced and included multiple synchronized display panels (Figure 1). The left panel showed time-series graphs of the drawing characteristics, including x- and y-position coordinates (0‐100 scale), pen pressure (0‐100 scale), pen altitude angle (14°‐44°), and pen azimuth direction (20°‐50°). The top middle panel displayed the video feed of the child, including facial expressions and gaze features. The bottom middle panel showed the actual drawing in real time. Each video also displayed the AI system’s risk assessment, categorized as either “low risk” (low ASD risk) or “high risk” (ASD risk present). Additional details are provided in Multimedia Appendix 1 [30,31].

The order of video presentation was not randomized because of the exploratory nature of the study; all participants viewed the videos in the same sequence. Participants were informed of the AI system’s previously reported diagnostic performance [21] and that it analyzed children’s shape-drawing processes to classify ASD risk. However, participants were not provided with detailed information about feature importance, explanations for specific classifications, training data characteristics, uncertainty estimates, or potential failure modes. Video 1 was 29 seconds long and video 2 was 50 seconds long. Participants could view the videos at their own pace using standard playback controls; individual viewing time data were not collected. Participants were instructed to make their own judgments while considering the AI output.

After viewing each video, participants completed a brief questionnaire about that specific case before proceeding to the next video. Each participant completed 2 trials, yielding 110 observations. After the video-viewing phase, participants rated their dispositional trust in AI-provided information and their agreement with the use of AI to assist or replace human professionals.

Participants completed an online survey administered through Microsoft Forms, which they could complete at their own pace with the option to pause and resume. The survey included informed consent procedures, demographic questions, the video-based assessment tasks, and trust-related questionnaire items. All survey items required a response, and each participant submitted the survey only once.

### Postvideo Questionnaire

The trust measurement items were adapted from established frameworks in human-AI interaction research [32,33]. Pilot testing with 2 ASD assessment professionals was conducted to verify face validity and comprehensibility in the context of AI-assisted ASD screening rather than to establish formal psychometric validation. The questionnaire included case-specific items administered after each video and general items administered at the end of the study. On the basis of the pilot feedback, the items were considered appropriate for an initial exploration of trust factors in this specialized professional population.

For each video, participants answered 3 questions. First, they provided a subjective assessment of ASD risk by selecting either “low risk” or “high risk.” Second, they rated confidence in their own assessment on a 7-point Likert scale ranging from 1 (not at all) to 7 (very much). Third, they rated their context-specific trust in the AI system’s decisions for that case on a 7-point Likert scale ranging from 1 (not at all) to 7 (very much). The primary dependent variable was participants’ context-specific trust in the AI system’s decisions.

General attitudes toward AI were assessed at the end of the study using 2 items. Participants rated their dispositional trust in AI-provided information on a 7-point Likert scale ranging from 1 (not at all) to 7 (very much). They also rated their agreement with the use of AI to assist or replace human professionals on a 7-point Likert scale ranging from 1 (strongly disagree) to 7 (strongly agree).

### Statistical Analysis

We analyzed the data using the *lme4* package in R (version 4.6.0; R Foundation for Statistical Computing). Descriptive statistics summarized the sample characteristics. We used a linear mixed model (LMM) to analyze the relationship between context-specific trust in AI decisions and participant characteristics while accounting for the nested data structure (multiple observations per participant). The dependent variable was context-specific trust in the AI system’s decisions for each case (on a 7-point scale). The model included participant ID as a random effect to account for individual differences. Trial (1 or 2) was also included as a random effect to account for potential order or learning effects.

Fixed effects included 6 participant-level predictors: age, years of professional experience, length of education required to obtain professional credentials, dichotomized frequency of AI application use (0=never or rarely, 1=several times per month, several times per week, or daily), dispositional trust in AI-provided information, and agreement with the use of AI to assist or replace human professionals. We also included confidence in participants’ own assessment as a trial-level covariate to examine its association with context-specific trust in the AI system. A 2-tailed binomial test compared the match rate between participants’ and the AI system’s judgments with the chance level. Statistical significance was set at α=.05 for all tests.

## Results

All participants completed the study without issues, and there were no missing data for the primary variables. As a result, all 110 observations were included in the final analyses. Participant characteristics are presented in Table 1.

**Table 1.** Participant demographics and professional characteristics (N=55).

Characteristic	Participants
Age (years), mean (SD)	44.3 (11.0)
Sex, n (%)	
Female	43 (78.2)
Male	11 (20)
Prefer not to answer	1 (1.8)
Profession, n (%)	
Clinical psychologist	15 (27.3)
Occupational or physical therapist	11 (20)
Public health nurse	9 (16.4)
Physician	5 (9.1)
Nurse	5 (9.1)
Nursery or kindergarten teacher	4 (7.3)
Early childhood education specialist	2 (3.6)
Combined roles	4 (7.3)
Highest level of education, n (%)	
Graduate school (doctoral)	4 (7.3)
Graduate school (master’s)	14 (25.5)
University (4 years)	23 (41.8)
Vocational school	9 (16.4)
Junior college	3 (5.5)
Higher technical college	2 (3.6)
Length of professional experience, (years) mean (SD)	19.4 (11.0)
Length of education required to obtain professional credentials (years), mean (SD)	4.6 (2.0)
Frequency of AI application use, n (%)	
Never used	8 (14.5)
Rarely used	14 (25.5)
Several times per month	14 (25.5)
Several times per week	14 (25.5)
Daily	5 (9.1)

All participants completed the items assessing general AI attitudes. Dispositional trust in AI-provided information was moderate (mean 3.73, SD 1.19), and agreement with the use of AI to assist or replace human professionals was slightly above the midpoint (mean 4.40, SD 1.27).

Across the 55 participants, the dichotomized frequency of AI application use indicated that 22 (40%) participants were nonfrequent users (never or rarely) and 33 (60%) participants were frequent users (several times per month to daily). Across the 110 observations, participants’ overall context-specific trust in the AI system’s decisions was moderate (mean 3.84, SD 0.95), with trial-specific values of mean 3.78 (SD 0.89) for video 1 and mean 3.89 (SD 1.00) for video 2. Conversely, participants’ overall confidence in their own assessment was relatively low (mean 2.45, SD 1.36), with mean 2.22 (SD 1.23) for video 1 and mean 2.69 (SD 1.44) for video 2. Subjective ASD risk assessments included 51.8% (57/110) instances of “high risk” and 48.2% (53/110) instances of “low risk.” The match rate between participants’ judgments and the AI system’s judgments was 51.8% (57/110), indicating near-chance agreement, which reflects the deliberately selected ambiguous cases. The match rate did not differ significantly from the chance level (2-tailed binomial test, *P*=.78). The primary aim was to examine the individual factors associated with trust rather than agreement rates.

We analyzed the data using an LMM (generalized *χ*^2^/df=0.108). Random-effects variance components are presented in Table 2. The results indicated significant between-participant (ID) variability in context-specific trust but no significant trial effect, with the trial variance component estimated near zero (Table 2). The substantial ID variance component suggests that individual differences accounted for a large proportion of variability in context-specific trust ratings.

**Table 2.** Random-effects parameter estimates from generalized linear mixed model predicting context-specific trust in AI system decisions (N=55; 110 observations).

	Estimate (SE)	95% CI	Wald *P* value
Participant ID	0.750 (0.083)	0.545-0.871	<.001
Trial	0.000 (0.056)	0.000-0.221	>.99
Residual	0.426 (0.042)	0.355-0.520	—^a^

a Not applicable.

Fixed-effects results are presented in Table 3. Both years of professional experience and the length of education required to obtain professional credentials were significantly and negatively associated with context-specific trust. Conversely, dispositional trust in AI-provided information demonstrated a significant positive association with trust ratings. Confidence in participants’ own assessment was significantly and positively associated with context-specific trust.

Effect size estimates were calculated following the method described by Nakagawa and Schielzeth [34]. The marginal *R*^2^=0.33 (variance explained by fixed effects alone), and the conditional *R*^2^=0.84 (variance explained by the combined fixed and random effects).

**Table 3.** Fixed effects parameter estimates from generalized linear mixed model predicting context-specific trust in AI system decisions (N=55; 110 observations).

	β^a^ (SE; 95% CI)	*t* test (*df*)	*P* value
Intercept	2.485 (0.748; 1.019 to 3.951)	3.32 (46.6)	.002
Age	0.024 (0.020; −0.015 to 0.062)	1.22 (46.0)	.23
Years of professional experience	−0.046 (0.021; −0.086 to −0.005)	−2.20 (46.3)	.03
Length of education required to obtain professional credentials	−0.171 (0.064; −0.296 to −0.046)	−2.67 (47.6)	.01
Frequency of use of AI applications (eg, ChatGPT)	−0.269 (0.250; −0.759 to 0.221)	−1.07 (45.9)	.29
Dispositional trust in AI-provided information	0.295 (0.106; 0.087 to 0.502)	2.78 (46.1)	.008
Level of agreement with the use of AI to assist or replace human professionals	0.063 (0.112; −0.156 to 0.282)	0.57 (45.9)	.57
Confidence in the assessment	0.301 (0.053; 0.197 to 0.405)	5.67 (91.0)	<.001

a β: unstandardized regression coefficient.

## Discussion

### Principal Findings

This study examined the relationship between trust in AI and professionals’ individual characteristics. Greater professional experience was associated with lower trust in AI, suggesting reluctance to adopt AI in clinical decision-making. Similarly, a longer period of education required for professional qualification was associated with reduced trust in AI. These findings suggest that practitioners with extensive clinical experience or longer credentialing pathways may apply stricter standards when evaluating AI outputs. Thus, lower context-specific trust among experienced professionals should not be interpreted solely as resistance to AI; it may also reflect calibrated skepticism rooted in deeper clinical knowledge and a more cautious evaluation of the correspondence between AI output and clinical judgment. By contrast, dispositional trust in AI-provided information was positively associated with AI use. Notably, even experienced professionals may be more likely to incorporate AI into their assessments when they are familiar with AI systems and trust their outputs. Furthermore, confidence in participants’ own assessment was positively associated with context-specific trust in AI, suggesting that professionals who feel more certain in their own judgments may also be more willing to align their trust with AI system decisions.

Previous studies in oncology have reported that more experienced clinicians tend to evaluate AI systems more critically [35,36]. Our findings suggest that a similar pattern may exist in ASD. Furthermore, dispositional trust in AI influences trust in domain-specific applications [10], consistent with our findings. These findings suggest that the individual factors influencing trust in AI in ASD may resemble those observed in other medical domains.

The pathophysiology and therapeutic mechanisms of ASD remain incompletely understood [37,38]. Professionals typically assess patients based on their education and clinical experience, as clinical expertise is central to medical decision-making [39], particularly in ASD. Reliance on AI systems may undermine clinical abilities [40]. Therefore, maintaining independent clinical decision-making while incorporating education on AI principles and strategies for effective human-AI collaboration into professional training is essential. This approach may support appropriate trust calibration and the effective integration of AI into clinical practice.

Recent theoretical frameworks on human-AI trust may help explain the present findings. The trustworthiness assessment model (TrAM) proposed by Schlicker et al [26] distinguishes between actual and perceived trustworthiness, suggesting that trust-related decisions are shaped by individual evaluative standards. Within this framework, experienced clinicians may apply more stringent evaluative criteria when assessing AI systems, potentially resulting in lower perceived trustworthiness despite stable objective performance. In addition, the concept of epistemic vigilance may help explain why professional expertise and a longer duration of education were associated with lower context-specific trust in AI systems in the present study. From this perspective, lower context-specific trust among experienced clinicians may reflect appropriately calibrated skepticism rather than simple resistance to AI technologies. Furthermore, Galindez-Acosta and Giraldo-Huertas [41] proposed the concept of deferred trust within the TrAM framework, highlighting how informational expertise and evaluative standards shape trust calibration toward AI systems. Together, these frameworks provide useful theoretical perspectives for interpreting the present findings.

Recent studies suggest that trust in AI-assisted decision-making is highly selective and context-dependent rather than solely a stable dispositional characteristic. Van Arum et al [33] demonstrated that context-specific trust in AI-assisted health decisions varies according to decision-making styles and the contextual appropriateness of AI outputs. From this perspective, clinicians’ context-specific trust in AI systems for ASD screening may represent a dynamic appraisal influenced by case complexity, diagnostic uncertainty, and the perceived fit between AI-generated information and clinical needs. Therefore, the ambiguous screening scenarios used in the present study may have elicited lower trust across participants, and trust responses may differ in more clinically straightforward situations. Future studies should examine the contextual factors and decision-making styles that influence selective trust in AI systems.

AI-based assessment in ASD is expected to continue advancing. Simultaneously, standardizing clinical assessment remains a critical challenge. Addressing these challenges will require professional education programs that integrate knowledge of AI principles and strategies for its appropriate use in clinical practice.

### Limitations

This study had several limitations. First, the sample size was relatively small, limiting statistical power to detect small individual-difference effects and the generalizability of the findings. Therefore, the findings should be interpreted as preliminary and exploratory. Larger studies are needed to confirm them. Second, we did not account for personality traits, which are established factors associated with trust in AI [30,42]. Future research incorporating personality assessments could clarify these relationships.

Third, although atypical fine motor and graphomotor skills, including handwriting and drawing difficulties, have been reported in children with ASD [43-48], ASD is a heterogeneous condition that involves multiple domains beyond these abilities. Therefore, reliance on a single geometric drawing task may limit the generalizability of the findings to broader ASD screening approaches. Future studies should use multimodal assessment tasks.

Fourth, the study design may have influenced participants’ trust ratings. Participants were not provided with detailed information about the AI system (eg, feature importance, training data, or failure modes). Although this approach reflects realistic clinical implementation, it may have influenced trust ratings. Future studies should examine trust calibration across different AI performance levels and include appropriate control conditions.

Fifth, several measurement-related limitations should be considered. Professional expertise was represented only by years of experience and duration of education, without assessing the quality of clinical training. AI familiarity was estimated based on the frequency of AI application use rather than validated measures of AI literacy [49,50]. Trust in AI was assessed using a single self-report item rather than behavioral measures of reliance, and the fixed presentation order may have introduced order effects that were not fully accounted for by the LMM.

Sixth, the findings should be interpreted within the Japanese cultural and professional context. Differences in health care systems, professional education, and attitudes toward AI may influence trust in AI-assisted diagnosis [51]. Therefore, caution is warranted when generalizing these findings to other countries.

### Conclusions

To our knowledge, this study is the first to examine the relationship between context-specific trust in AI systems for ASD screening and professionals’ individual characteristics. Our findings suggest that more experienced clinicians tend to exhibit greater skepticism toward AI, consistent with observations in other medical fields, such as oncology. To support effective integration of AI into clinical practice, professional training should include education on AI principles and strategies for effective human-AI collaboration. Future studies with larger samples and more detailed measures are needed to further clarify these relationships.

## Supplementary material

10.2196/98367 Multimedia Appendix 1 Trust measurement items.

## Abbreviations

ASD: autism spectrum disorder
CHERRIES: Checklist for Reporting Results of Internet E-Surveys
LMM: linear mixed model
STROBE: Strengthening the Reporting of Observational Studies in Epidemiology
TrAM: trustworthiness assessment model
