# Supplementary material for: Factors Associated With Professionals’ Context-Specific Trust in AI-Based Screening for Autism Spectrum Disorder: Cross-Sectional Survey Study
Source: JMIR Form Res. 2026 Sep 25;10:e98367. doi: 10.2196/98367 (PMC13614214; doi:10.2196/98367)
Supplement: Supplementary file 1 [file formative-v10-e98367-s001.docx]

# Supplementary File: Trust Measurement Items

## Overview

This supplementary file provides the complete questionnaire items in Japanese and English, along with a mapping between each key item and the corresponding constructs or scales in prior literature.

## Participant-Level Items

| **No.** | **Japanese** | **English** | **Response Format** |
| --- | --- | --- | --- |
| 1 | 専門職の経験年数をご回答ください。 | Please indicate your years of professional experience. | Numeric (years) |
| 2 | 最終学歴をご回答ください。 | Please indicate your highest level of education. | Categorical (e.g., Bachelor's, Master's, PhD) |
| 3 | ChatGPTやGemini，Claudeなどの人工知能（大規模言語モデル）をどれぐらいの頻度で使用していますか？ | How frequently do you use AI applications (e.g., ChatGPT, Gemini, Claude)? | 5-point Likert scale: 4 (Every day) / 3 (Several times a week) / 2 (Several times a month) / 1 (Rarely) / 0 (Never) |
| 4 | AIが提供する一般的な情報について、どの程度の信頼がありますか？ | To what extent do you trust general information provided by AI? | 7-point Likert scale: 1 (Not at all) to 7 (Very much) |
| 5 | AIが人間の専門職を補助・代替することに賛成ですか？ | Do you agree with the use of AI to assist or replace human professionals? | 7-point Likert scale: 1 (Strongly disagree) to 7 (Strongly agree) |

## Trial-Level Items (After Each Video)

| **No.** | **Japanese** | **English** | **Response Format** |
| --- | --- | --- | --- |
| 6 | ビデオを見た結果、あなたの主観的な判断では、ASDリスクはどちらと考えますか。 | Based on the video, what is your subjective judgment of ASD risk? | Binary choice: Low risk / High risk |
| 7 | あなたはご自身の判断にどの程度自信がありますか。 | How confident are you in your own judgment? | 7-point Likert scale: 1 (Not at all) to 7 (Very much) |
| 8 | ビデオを見た結果、このAIシステムの判断はどの程度信頼できると思いますか。 | Based on the video, to what extent do you trust this AI system’s judgment? | 7-point Likert scale: 1 (Not at all) to 7 (Very much) |

## Correspondence Table: Key Items and Source Literature

The following table focuses on the main items analyzed in this study (Items 4, 5, 7, and 8) and summarizes how they conceptually relate to the constructs and scales discussed in Riedl (2022) and Schepman and Rodway (2023).

| **Item in this study** | **Construct / sub-construct** | **Reference** | **Scale / subscale in reference** | **Relation to the present item** |
| --- | --- | --- | --- | --- |
| **Item 4** (Trust in information provided by AI) | Dispositional trust in AI systems; dispositional trust in automation/machines | Riedl (2022) [1] | Disposition to trust; dispositional trust in automation/machines (reviewed as specific personality traits) | Item 4 is a single-item indicator of how much participants generally trust information produced by AI systems, conceptually grounded in Riedl's discussion of dispositional trust and dispositional trust in automation/machines as antecedents of trust in AI. |
| **Item 4** (Trust in information provided by AI) | General attitudes toward AI, including usefulness, performance, and error concerns (reliance-based trust) | Schepman and Rodway (2023) [2] | General Attitudes towards Artificial Intelligence Scale (GAAIS): Positive and Negative subscales | Item 4 summarizes, in a single 7-point item, the extent to which participants feel they can rely on AI-generated information, drawing on the trust-related facets covered by the GAAIS (performance, usefulness, and error concerns). |
| **Item 5** (Attitudes toward AI assistance / replacement) | Acceptance and adoption of AI systems in professional contexts | Riedl (2022) [1] | Trust in AI systems as an antecedent of acceptance and adoption (theoretical framework) | Item 5 operationalizes, in the specific context of ASD screening and other professional tasks, the degree to which participants accept or oppose the introduction of AI that assists or replaces human professionals, in line with Riedl's acceptance/adoption perspective. |
| **Item 5** (Attitudes toward AI assistance / replacement) | Positive attitudes towards AI in work and society (augmentation / replacement) | Schepman and Rodway (2023) [2] | GAAIS Positive subscale (work- and job-related items) | Item 5 is a direct, single-item formulation of attitudes toward AI augmenting or replacing human professionals, conceptually linked to the job- and society-related items in the GAAIS Positive subscale. |
| **Item 7** (Confidence in own judgment) | Task-specific self-efficacy in AI-supported decision-making; internal locus of control | Riedl (2022) [1] | Specific personality traits reviewed: self-efficacy, locus of control | Item 7 is a newly created single item that extends Riedl's discussion of self-efficacy and internal locus of control into the ASD screening context, measuring, on a trial-by-trial basis, how confident participants are in their own judgment when evaluating ASD risk alongside AI. |
| **Item 7** (Confidence in own judgment) | – | Schepman and Rodway (2023) [2] | – | There is no direct scale counterpart for Item 7 in the GAAIS. The item is conceptually complementary to the GAAIS: whereas the GAAIS measures attitudes toward AI, Item 7 captures human-side self-confidence in decision-making when AI is present. |
| **Item 8** (Context-specific trust in the AI agent's judgment) | Trust in a specific AI system in a given context (ASD screening); trusting beliefs about performance, functionality, predictability, and reliability | Riedl (2022) [1] | Trusting beliefs in human–technology relationships: performance, functionality, helpfulness, predictability, reliability | Item 8 is a global 7-point evaluation of how much participants trust the ASD-screening AI agent's judgment for each video, integrating multiple trust facets (performance, reliability, predictability) into a single context-specific trust rating for a specific AI system. |
| **Item 8** (Context-specific trust in the AI agent's judgment) | Reliance-based trust and safety; controllability and perceived danger of AI | Schepman and Rodway (2023) [2] | GAAIS Negative subscale (trust-related items) | Item 8 corresponds to a context-dependent, single-item measure of how much participants are willing to rely on the ASD-screening AI agent's judgment in each trial, drawing on the reliance-, controllability-, and danger-related facets of trust captured by the GAAIS Negative subscale but applied to a specific AI system and task. |

## References

1. Riedl R. Is trust in artificial intelligence systems related to user personality? Review of empirical evidence and future research directions. Electron Mark. 2022;32(4):2021–2051. doi: 10.1007/s12525-022-00594-4

2. Schepman A, Rodway P. The **g**eneral **a**ttitudes **t**owards **a**rtificial **i**ntelligence **s**cale (GAAIS): **c**onfirmatory validation and associations with personality, corporate distrust, and general trust. *Int J Hum Comput Interact.* 2023;39(13):2724–2741.

https://doi.org/10.1080/10447318.2022.2085400
